# Bioactive *Azadirachta indica* and *Melia azedarach* leaves extracts with anti-SARS-CoV-2 and antibacterial activities

**DOI:** 10.1371/journal.pone.0282729

**Published:** 2023-03-08

**Authors:** Bahaa A. Hemdan, Ahmed Mostafa, Marwa M. Elbatanony, Amal M. El-Feky, Tsvetelina Paunova-Krasteva, Stoyanka Stoitsova, Mohamed Azab El-Liethy, Gamila E. El-Taweel, Mobarak Abu Mraheil

**Affiliations:** 1 Water Pollution Research Department, Environmental Microbiology Laboratory, National Research Centre, Dokki, Cairo, Egypt; 2 Center of Scientific Excellence for Influenza Viruses, National Research Centre, Dokki, Cairo, Egypt; 3 Pharmacognosy Department, National Research Centre, Dokki, Cairo, Egypt; 4 The Stephan Angeloff Institute of Microbiology, Bulgarian Academy of Sciences, Sofia, Bulgaria; 5 Institute of Medical Microbiology, German Center for Infection Research (DZIF), Partner Site Giessen-Marburg-Langen Site, Justus-Liebig University Giessen, Giessen, Germany; Universidad Autonoma de Chihuahua, MEXICO

## Abstract

The leaves of *Azadirachta indica* L. and *Melia azedarach* L., belonging to Meliaceae family, have been shown to have medicinal benefits and are extensively employed in traditional folk medicine. Herein, HPLC analysis of the ethyl acetate fraction of the total methanolic extract emphasized the enrichment of both *A*. *indica* L., and *M*. *azedarach* L. leaves extracts with phenolic and flavonoids composites, respectively. Besides, 4 limonoids and 2 flavonoids were isolated using column chromatography. By assessing the *in vitro* antiviral activities of both total leaves extracts against Severe Acute Respiratory Syndrome Corona virus 2 (SARS-CoV-2), it was found that *A*. *indica* L. and *M*. *azedarach* L. have robust anti-SARS-CoV-2 activities at low half-maximal inhibitory concentrations (IC_50_) of 8.451 and 6.922 μg/mL, respectively. Due to the high safety of *A*. *indica* L. and *M*. *azedarach* L. extracts with half-maximal cytotoxic concentrations (CC_50_) of 446.2 and 351.4 μg/ml, respectively, both displayed extraordinary selectivity indices (SI>50). *A*. *indica* L. and *M*. *azedarach* L. leaves extracts could induce antibacterial activities against both Gram-negative and positive bacterial strains. The minimal inhibitory concentrations of *A*. *indica* L. and *M*. *azedarach* L. leaves extracts varied from 25 to 100 mg/mL within 30 min contact time towards the tested bacteria. Our findings confirm the broad-spectrum medicinal value of *A*. *indica* L. and *M*. *azedarach* L. leaves extracts. Finally, additional *in vivo* investigations are highly recommended to confirm the anti-COVID-19 and antimicrobial activities of both plant extracts.

## 1. Introduction

Drug resistance to infections in humans brought on by microorganisms including bacteria, viruses and fungi is a life-endangering phenomenon with considerable number of mortalities worldwide. Medicinal herbs were applied to produce a plenty of pharmaceuticals that are currently used to alleviate various health disorders or diseases. Numerous researches are performed to find out new antimicrobial treatment alternatives that really can prevent the spread of bacteria or eradicate them without endangering humans by conducting comprehensive screenings of medicinal herbs [1, 2].

In December 2019, the SARS-CoV-2 virus has emerged as an unknown severe respiratory illness, namely corona virus illness 2019 (COVID-19) [3]. Few weeks after the spark of infection in China in 2019, the infections with SARS-CoV-2 expanded globally to all continents, urging WHO to declare COVID-19 as an international concern and further as a global pandemic in early 2020 [4]. Since the establishment of COVID-19 pandemic, FDA (Food and Drug Administration)-approved drugs and medicinal plants exhibiting broad antiviral effects were ranked first as a safe tool to be brought into the antiviral discovery programs for such pandemic [5–7]. For centuries, herbal products have indeed been utilized to control a diversity of contagious diseases because of their powerful antimicrobial, anticancer, and anti-inflammatory capabilities and their surprisingly low risk of health consequences [8].

*Azadirachta indica* L. (Neem) and *Melia azedarach* L. (China tree) are popular medicinal herbs that belong to the Meliaceae family. *A*. *indica* L, originated in India, is extensively scattered in all countries [9]. Various phytoconstituents were identified from neem leaves such as triterpenoids, phenolics, flavonoids, carotenoids and steroids, that proved to be significant in many biological fields especially antibacterial, antifungal, antiviral, antiparasitic, and many chronic diseases as well [10]. Limonoids, a class of triterpene and a major class identified from the Meliaceae family including different compounds and their derivatives (e.g. nimbidin, nimbin, and nimbolide) are broadly circulated all over the plant parts [11]. Through changes in several genetic pathways and other effects, they recreate a significant function in regulating human ailments [12]. A *indica* has great medicinal values and was traditionally utilized to treat stomach ulcers and jaundice besides its significance in controlling a variety of infectious diseases such as malaria [13]. Lately, limonoids are evidenced to have curative role in digestive system complaints and as an insects repellent, in addition to its significant effect as diuretic and anti-diabetic, and healing numerous skin diseases as well [14].

On the other hand, *M*. *azedarach* is native to Africa, Asia and Northern Australia, and is conventionally employed as an antiparasitic and antifungal agent with significant free radical scavenging activity [15, 16]. The bioactivity of such herbal plant is principally accredited to occurrence of high level of limonoids compounds besides other phenolic acids and flavonoid glycosides mainly rutin [11, 17]. *M*. *azedarach* L. leaves contain significant quantities of limonoids of the nimbolinin type, which have a variety of therapeutic effects, including antimicrobial, antioxidant, and anticancer potential [18].

A lot of emphasis has indeed been devoted to the investigation for antimicrobial compounds in sustainable materials, and efforts have been taken to find compounds that can adequately replace manufactured antimicrobials. Plant-based phytochemicals are employed as a paradigm to generate healthier and more efficacious therapeutics for preventing the growth of microorganisms. Different plants extracts have been utilized in various researches to screen for antimicrobial property and discover novel antimicrobial substances [24]. Both *A*. *indica* L. and *M*. *azedarach* L., belonging to Meliaceae family, were documented to have inhibitory effect against bacteria due to their enrichment in antimicrobial bioactive compounds [19, 20]. They have been widely used in traditional Chinese and Ayurvedic prescriptions to control various infections [21]. *A*. *indica* L., Aside from the plant’s phytochemistry, a lot of development has been made over the past 20 years in terms of antimicrobial activity and prospective medical uses [22].

The main active chemical ingredients of Neem leaves are nimbin, nimbanene, 6-desacetylnimbinene, nimbandiol, nimbolide, ascorbic acid, n-hexacosanol aminoacid, 7-desacetyl-7-benzoylazadiradione, 7-diacetyl-7-benzoylgedunin, 17-hydroxyazadiradione and nimbiol [23]. Traditionally, Neem leaves extract are utilized in folk medicine to prevent infectious bacterial and parasitic diseases. In addition, researchers reported many other biological and pharmacological activities including antiviral, antifungal, anti-inflammatory, antipyretic, antiseptic, and cytotoxic activities [19].

Herein, we evaluated the anti-SARS-CoV-2 and antibacterial activities of *A*. *indica* L. (Neem) and *M*. *azedarach* L. leaves ethyl acetate fractions and evaluating their phenolic profile against pandemic SARS-CoV-2 virus and some selected pathogenic bacterial strains.

## 2. Materials and methods

### 2.1. Chemicals and plant materials

All solvents utilized in this current study were of analytical grade (BDH, UK and Merck, Germany). Fresh leaves of *A*. *indica* L., and *M*. *azedarach* L. were obtained from Orman botanical garden, Giza, Egypt and identified by Mrs. Trease Labib specialist of plant taxonomy. Leaves were immediately wrapped in plastic packaging after becoming air-dried.

### 2.2. Extraction procedure

Extraction was performed using cold extraction method [24]. Briefly, the air-dried leaves of *A*. *indica* L., and *M*. *azedarach* L. (500 mg) were separately soaked with 80% methanol solvent for 72 h (statically, 2Lx5 at room temperature). Subsequently, the extract was filtered on gauze and the used solvent was vanished entirely using rotary evaporator instrument. The leftover residue was then suspended in double distilled water (ddH_2_O). Extract was moved into a separating funnel to complete the defatting process using petroleum ether (40–60°C) and fractionation using ethyl acetate repeatedly [25]. Further, the obtained fractions were concentrated by using rotary evaporator under 40°C till further phytochemical investigations.

### 2.3. HPLC analysis of the ethyl acetate fraction

To characterize the chemical composition of the ethyl acetate fraction, HPLC was done using Agilent Technologies 1100 series liquid chromatograph that is supplemented with an auto sampler and a diode-array detector (DAD). Eclipse XDB-C18 (150 x 4.6 μm; 5 μm) analytical column with a C18 guard column was used (Phenomenex, Torrance, CA). Acetonitrile (solvent A) and 2% acetic acid in water (v/v) made up the mobile phase (solvent B). The gradient was programmed as follows: 0–5 min, 100% B (isocratic step); 30 min, 100–85% B (linear gradient); 20 min, 85–50% B (linear gradient); 5 min, 50–0% B (linear gradient); 5 min, 0–100% B (linear gradient) at a flow rate of 0.8 ml/min. Peaks for the benzoic acid, cinnamic acid derivatives, and flavonoids were sequentially analyzed at 280, 320, and 360 nm for the injection volume of 20 L, correspondingly. Prior to injection, all specimens were purified with an Acrodisc 0.45 μm syringe filter (Gelman Laboratory, Michigan, USA). Peaks were identified by correlating UV spectra and retention times, and these were then compared with the standards that run under the same conditions.

### 2.4. Isolation of major compounds

The ethyl acetate fraction (4 g) of both plant leaves was subjected to silica gel column chromatography (Silica gel 60, Merck, Germany) eluted using 100% *n-*hexane, and different ratios of mixture of ethyl acetate, and methanol. The collected fractions were evaporated under vacuum and examined by thin layer chromatography (TLC) which was carried out on pre-coated silica gel 60 F254 TLC plate (Merck). The plates were visualized under UV at λmax-254 nm and spraying with 10% sulfuric acid and AlCl_3_. Similar fractions were combined together according to R_*f*_ values. Detailed structure elucidation of the isolated compounds were performed by different spectral analyses (IR, mass spectrometry, and ^1^H-NMR (400 MHz), and ^13^C-NMR (125 MHz)).

### 2.5. Anti-SARS-CoV-2 virus activity

#### 2.5.1. Cell lines and viruses

Vero-E6 monkey kidney cell line was cultivated in growth Dulbecco’s Modified Eagle’s medium (DMEM, Lonza), complemented with 1% Penicillin/Streptomycin mixture (pen/strep) and 10% Fetal Bovine Serum (FBS) (Invitrogen, Germany). Cultured cell line was kept in a humidified 5% CO_2_ incubator at 37°C. The hCoV-19/Egypt/NRC-3/2020 SARS-CoV-2 virus (GISAID accession number: EPI_ISL_430820) [26], was propagated in Vero-E6 cells to prepare virus stock as described previously [27]. The propagated viral stock was then purified by centrifugation, aliquoted, titrated and stored in a -80°C freezer until further use.

#### 2.5.2. Cytotoxic concentration 50 (CC_50_) determination

Solution of the examined plant extracts were established in 10% DMSO and diluted into serial dilutions with DMEM (1 μg/ml– 10 mg/ml) to estimate the half maximum cytotoxic concentration (CC_50_). The cytotoxic effectiveness of the extracts was investigated utilizing 3-(4, 5-dimethylthiazol-2-yl)-2, 5-diphenyltetrazolium bromide (MTT) assay with minimal alterations in Vero E6 cells, an immortalized cell line generated from renal epithelial cells of the African green monkey [5, 6, 28]. We employed non-linear regression data to compare the cytotoxicity of differing extract concentrations on treated to untreated cells by graphing log inhibition against standardized responsiveness.

#### 2.5.3. Inhibitory concentration 50 (IC_50_) determination

The IC_50_ values for the examined extracts were determined as previously described [5, 6]. Briefly, Vero-E6 cells were dispensed evenly into the 96-well tissue cultivation plates and were cultured overnight at 37°C with 5% CO_2_ to reach 80–90% confluency. The cell monolayers were then exposed to pre-incubated sample/virus mixtures containing 100 TCID50 (Median Tissue Culture Infectious Dose) of the virus. The treated cell monolayers are kept the mixtures containing different sample concentrations (<CC_50_) for 1 h at ambient temperature and then washed out with 1x phosphate buffer saline (PBS). Subsequently, 100 μl of infection media DMEM supplemented with 1% pen/strep and 0.2% Bovine Serum Albumin (BSA) were added. The cells were then kept for 72 h at 37°C in an incubator with 5% CO_2_. Eventually, cell monolayers were fixed with 100 μl of 4% para-formaldehyde for 20 minutes, stained further with 0.1% crystal violet in distilled water at RT for 15 min and destained with absolute methanol. The absorption intensity of the solubilized crystal violet color was then measured at 570 nm using an AnthosZenyth 200rt plate reader. In comparison to the virus and cell controls, the compound’s 50% inhibitory concentration (IC_50_) was calculated using GraphPad prism software by plotting the log concentration against normalized responses.

### 2.6. Antibacterial activities of prepared plant extracts

The bactericidalpotential of 50μg/mL of *A*. *indica* L. and *M*. *azedarach* L. leaves extracts were evaluated using the disc and well-diffusion assays by the formation of inhibition zone. Then, the broth-microdilution approach was used to estimate the values of minimum inhibitory concentration (MIC).

#### 2.6.1. The used bacterial strains and their preparation

The antibacterial actions of extracts were examined against *Escherichia coli* (ATCC 25922), *Salmonella enterica* (ATCC 14028), *Pseudomonas aeruginosa* (ATCC 10145), representing Gram-negative (GN) bacteria; *Listeria monocytogenes* (ATCC 35152), *Staphylococcus aureus* (ATCC 43300) and *Enterococcus faecalis* (ATCC 43845), representing Gram-positive (GP) bacteria. Each bacterial culture stock preserved in -80°C was injected into a tube containing 20 ml of Trypticase soya broth (TSB). Then, all inoculated tubes were incubated for 24 h at 37°C. Dilution of the cultures with Mueller–Hinton Broth (MHB) was performed corresponding to ten folds serial dilution upto 10^-8^CFU/ml of the bacterial culture.

#### 2.6.2. Agar dilution methods (well and disc diffusions

*2*.*6*.*2*.*1*. *Disc-diffusion method*. The technique was conducted based upon the Clinical and Laboratory Standards Institute (CLSI) (M02-A11) [29]. The prepared bacterial inoculum suspension was briefly swabbed onto MHA medium with a sterile cotton swab. Next, sterile discs made of filter paper (6 mm) (Maidstone, UK) were saturated with 50μg/mLof the tested samples. Both Ciprofloxacin (5μg/disc) and Vancomycin (30μg/disc) were used as controls. The saturated discs were lightly pushed on the top surfaces MHA and incubated with bacteria overnight at 37°C. A produced zone of inhibition (ZOI) was measured and given in millimeters (mm) [15].

*2*.*6*.*2*.*2*. *Well-diffusion method*. Using sanitized needle tips, a 6 mm-diameter hole was drilled. Afterward when, a sterilized cotton swab was employed to smear the inoculum standard solution of the examined microbes onto the medium. The specimen was then transferred to each well in intervals of 50 μL. Two reference antibiotic drugs (vancomycin and ciprofloxacin) served as positive controls. Filtered water functioned as the negative control. All inoculation plates were then incubated at 37°C on the plate. For the tested microorganisms, the inhibition areas were observed within 24 h [16].

#### 2.6.3. Broth-dilution assay and estimation of MICs values

The minimum inhibitory concentration (MIC) for antibacterial activity of leaves extracts of *A*. *indica* L. and *M*. *azedarach* L. were assessed against the above mentioned microbial pathogens. Briefly, 100 μl of each fresh grown microbial culture was inoculated into10 mL sterile distilled water (dH_2_O) tubes containing different concentrations (25, 50, 75, and 100 mg/ml) of the tested leaves extracts. In contrast, the control tubes for each tested strain were free from the tested extracts. The specific conditions of all trials were at ambient temperature (25±0.43°C) and 250 rpm of agitation in shaker. Samples were collected from each tube at four different time intervals (5, 10, 20, and 30 min). The bacterial cell densities were counted before (initial count) and after exposure to the tested extracts. The viable bacterial counts were calculated as CFU/mL for 24 h at 37°C using the pour plate method according to APHA [30]. All experimental trials were accomplished in three times intervals and repeated two times on dissimilar periods [17].

### 2.7. Statistical analysis

The inhibitory concentration (IC_50_) of leaf extracts of *A*. *indica* L. and *M*. *azedarach* L. was calculated using GraphPad Prism version 9.2.0. Moreover, the Minimum Inhibitory Concentration (MIC) of the two plant extracts against the tested bacterial strains was also computed using a two-way analysis of variance (*ANOVA*), where (**) denotes a moderate correlation (p ≤0.01) and (***) denotes a high correlation (p≤ 0.001). Additionally, the Person (r) correlation P-Value and R2 value between various plant extract doses and the examined bacterial strains were calculated.

## 3. Results and discussion

### 3.1. HPLC-DAD analysis

HPLC-DAD analysis was used to recognize and estimate phenolics and flavonoids in ethyl acetate fraction of the methanolic extract of *A*. *indica* L. and *M*. *azedarach* L. (**Table 1**). The analysis discovered the existence of 10 and 11 phenolics in *A*. *indica*, L. and *M*. *azedarach* L., respectively. Catechin was proved to be the dominant compound in both fractions (315.404 μg/g in *A*. *indica* L. and 198.247 μg/g *M*. *azedarach* L.), followed by vanillic acid (151.947 μg/g) in *A*. *indica* L. and chlorogenic acid (112.869 μg/g) for *M*. *azedarach* L. Additionally, four flavonols were identified in both fractions of which rutin was the major (**Table 1**).

**Table 1:** Polyphenolic compounds content from HPLC analysis of *A*. *indica* L. and *M*. *azedarach* L. leaves ethyl acetate fractions.

Compound		Amount (μg/gm)	
		*A*. *indica* L.	*M*. *azedarach* L.
Phenolic acids/compounds	Gallic acid	32.715	27.214
	Protocatechuic acid	59.866	27.415
	*p*-hydroxybenzoic acid	52.587	35.741
	Gentisic acid	0.000	99.581
	Catechin	315.404	198.247
	Chlorogenic acid	99.167	112.869
	Caffeic acid	50.341	17.104
	Syringic acid	8.979	15.889
	Vanillic acid	151.947	8.171
	Ferulic acid	13.592	36.934
	Cinnamic acid	8.869	15.290
Flavonols	Rutin	4150.625	1418.941
	Quercetin	38.274	45.908
	Kaempferol	25.713	19.233
	Chrysin	27.362	12.406

It is to be mentioned that phenolics and flavonoids are the dominant bioactive constituents in plant kingdom possessing various medicinal properties such as antioxidant, cytotoxic, anti-inflammatory and antimicrobial activities [24, 25]. The prime mechanism of action of attained phenolics and flavonoids is attributed to phenolic–protein interactions where their metabolites, which are present in a rather high concentrations, form the complex with functional enzymes and exert a clear radical scavenging action leading to developing their bioactivities [31].

### 3.2. Isolation and characterization of *A*. *indica* L. compounds

Column chromatography technique afforded the isolation of three compounds. Compounds 1 and 2 were isolated from 100% *n-*hexane fraction while compound 3was isolated from ethyl acetate: methanol (60:40 v/v).

**Compound 1:** white amorphous powder, m.p.: 180°C. IR data (KBr/ cm^-1^) = 3310 (OH str.), 2930(CH_2_& C-H str.), 1762 (C = O conjugation), 1653(C = C str.), 1445 (C-O-H str.), 1118 (C-O-C str.). EI-MS (*m*/*z*): 468(M+) for C_28_H_36_O_6_, 450 [M^+^ -H_2_O], ^1^H-NMR (CD_3_OD, *δ* / ppm): 7.11 (1H, *d*, H-1), 5.45 (1H, *d*, H-2), 2.89 (1H, *d*, H-5),4.29 (1H, *d*, H-5), 4.89(1H, *dd*, H-6), 4.95(1H, *d*, H-7), 2.67 (1H, *m*, H-9), 1.91(4H, *m*, H-11, H-12), 1.64 (1H, *s*, H-15), 7.39 (1H, *t*, H-21), 6.21 (1H, *s*, H-22), 6.93 (1H, *s*, H-23), 1.70 (1H, s, 6-OH), 2.15 (1H, s, AC), 1.10 (3H, s, C-methyls). ^13^C-NMR (CD_3_OD, *δ* / ppm): 158.2 (C-1), 126.4 (C-2), 206.1 (C-3), 41.3 (C-4), 50.4 (C-5), 69.8 (C-6), 76.9 (C-7), 43.7 (C-8), 40.2 (C-9), 41.3 (C-10), 16.1 (C-11), 28.3 (C-12), 45.7 (C-13), 72.8 (C-14), 57.5 (C-15),33.1 (C-16), 40.2 (C-17), 122.8 (C-20), 141.8 (C-21), 111.2 (C-22), 138.7(C-23), 21.51, 171.81 (OAC), 21.9 (C-methyls). From the formentioned spectral data, this compound was identified as 14,15-β-epoxynimonol [32].

**Compound 2:** amorphous powder, IR data (KBr/ cm^-1^) = 2913(CH_2_ and C-H str.), 1655 (C = C str.), 1722 (C = O), 1462 (C-O-H str.), 1063 (C-O-C str.). ESI-MS (m/z) showed [M]^+^ 612 for C_35_H_48_O_9_, 549 (100%), 449, 389 and 371. Additionally, ^1^H-NMR, and^13^C-NMR data were in agreement with previous studies [33], so that the compound was established as 1*α*-hydroxyl-3*α*-acetyl-7*α*-tigloyl-12*β*-ethoxyl-nimbolinin (12-ethoxynimbolininH)which was previously isolated from the bark of other *Melia* species [33, 34].

**Compound 3:** yellowish green powder; m.p. 166°C. IR data (KBr/ cm^-1^):3215 (OH str.), 2925(CH2 and C-H str.), 1710 (C = O conjugation), 1663(C = C str.), 1442 (C-O-H str.), 1123 (C-O-C str.). EI-MS (*m*/*z*): 432 (M+) for C_21_H_20_O_10_, 305, 245, 184, 170, 153, 125. ^1^H-NMR, ^13^C-NMRand UV spectral data with different shift reagents were in accordance with previous study [35], confirming that compound was genistein 7-*O*-glucoside.

### 3.3. Characterization of *M*. *azedarach* L. compounds

Column chromatography technique afforded the isolation of three compounds. Two compounds (4 and 5) from 100% n- hexane fraction and compound 6 eluted from ethyl acetate/Methanol (80:20 v/v).

**Compound 4:** amorphous powder. ESI-MS (m/z) showed [M]^+^ 694 for C_40_H_54_O_10_, 649 for ethoxyl group loss at C-12, then 449 for two tigloyl side chain loss, followed by 389(100%) due to acetyl group loss and subsequently water loss giving 371 which are in accordance to nimbolinin limonoids fragmentation pattern. The ^1^H and ^13^C NMR data were similar to [36], thus, the structure was elucidated as 1α,7α-ditigloyloxy-3α-acetoxyl-12α-ethoxylnimbolinin.

**Compound 5:** white amorphous powder. ESI MS (*m/z*) 554 [M] ^+^, 552, 537 [M–H_2_O + H]^+^. The data were consistent with the molecular formula C_35_H_54_O_5_. The ^1^H-NMR and^13^C-NMR data confirmed this compound as 3-α-tigloylmelianol which was previously identified from *M*. *azedarach* fruits by [37] but it is the first time to be detected in leaves.

**Fig 1:**
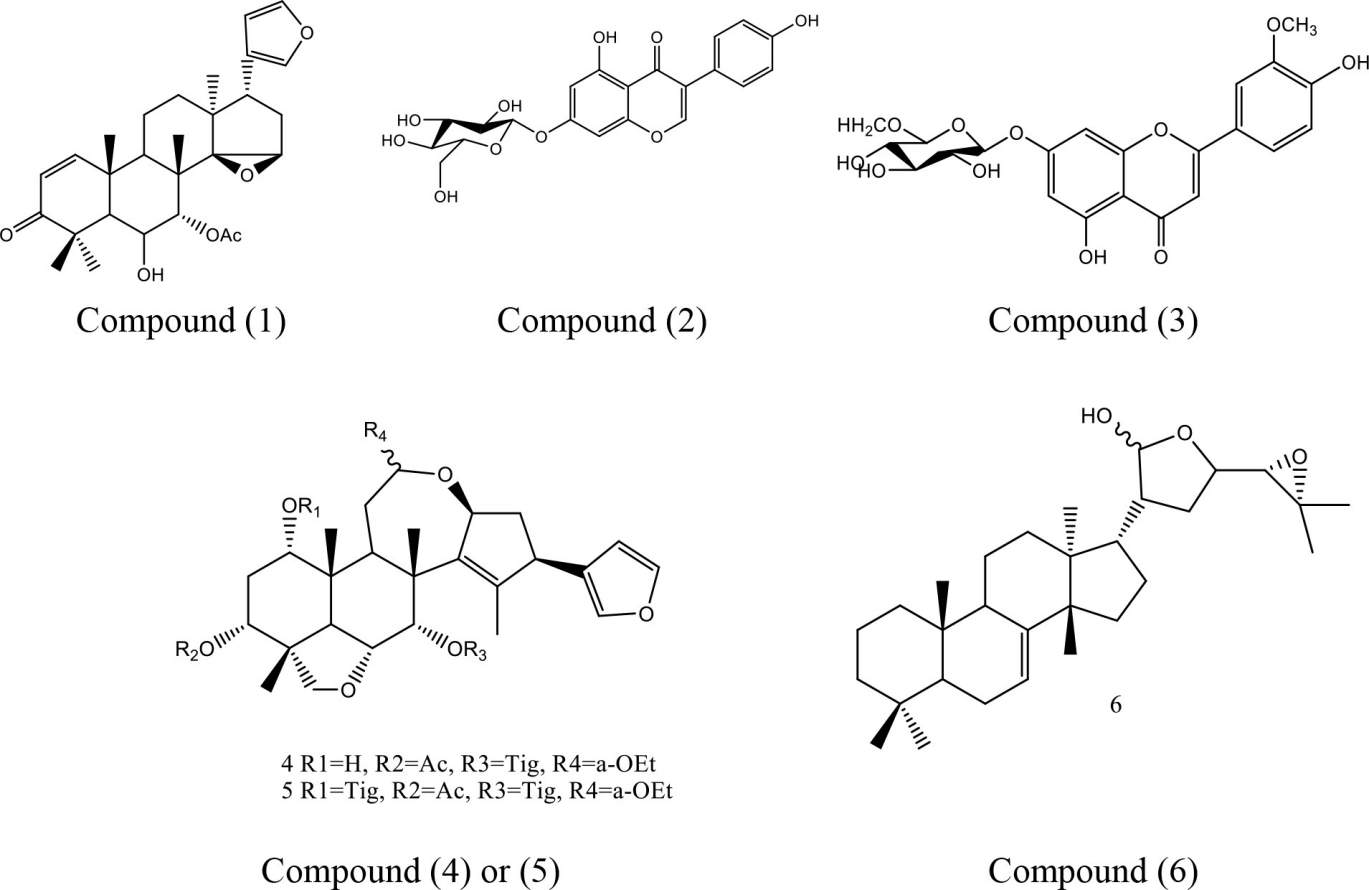
Structure of the isolated compounds from *A*. *indica* L. (compounds 1, 2, and 3) and from *M*. *azedarach* L. (compounds 4, 5, and 6).

**Compound 6:** yellow amorphous powder, m.p. 164°C. EI-MS (*m*/*z*): 462 (M+) for C_22_H_22_O_11_, in addition to 300, 285, 257, 152, 137, 116. IR data, UV absorbance in different shift reagents and ^13^C-NMR data characterized this compound as chrysoeriol 7-*O*-glucoside. This compound has been isolated for first time in the present study. The structures of the isolated compounds were illustrated in **Fig 1**.

### 3.4. Anti-SARS-CoV-2 virus activity

**Fig 2:**
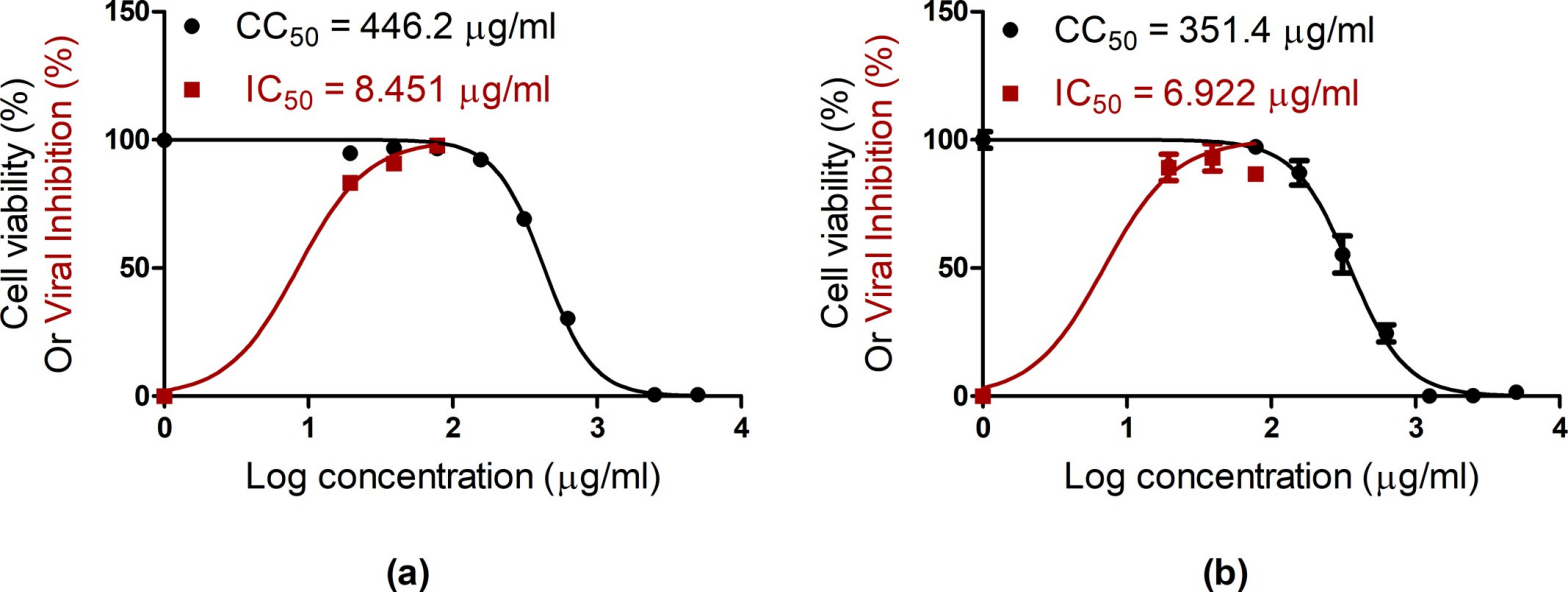
Cytotoxicty and anti-SARS-CoV-2 activities for *A*. *indica* L. (Neem) leaves extract (a) and *M*. *azedarach* L. leaves extract (b). CC_50_: half-maximal cytotoxic concentration; IC_50_: half-maximal inhibitory concentration; IS: selectivity index.

To appraise the antiviral efficacy of leaves extracts against SARS-CoV-2 virus, the half maximal cytotoxic concentration (CC_50_), the half maximal inhibitory concentration 50 (IC_50_) and selectivity index (SI)(CC_50_/IC_50_) against SARS-CoV-2 were individually evaluated (**Fig 2 and Table 2**). Both leaves extracts of *A*. *indica* and *M*. *azedarach* showed high safety in Vero E6 cells with CC_50_ values of 446.2 μg/mL for *A*. *indica* and 351.4 μg/mL for *M*. *azedarach*, respectively. Additionally, both extracts exhibited promising inhibition of SARS-CoV-2 replication in Vero E6 cells at potent IC_50_ concentrations of 8.541 μg/mL for *A*. *indica* and 6.922 μg/mL for *M*. *azedarach* (**Fig 2**). Interestingly, both extracts exhibited extraordinary selectivity indices (SI) (>50) (**Table 2**). The determined SI values are meaningfully greater than those recommended for further assessment of the bioactive compounds (> 10) [38, 39]. The SI values of 52.8 and 50.8 for *A*. *indica* and *M*. *azedarach* indicate the bioactive potential of *A*. *indica* and *M*. *azedarach* leaves extracts. Compared to the reference drug control remedesivir (CC_50_ = 473.1 μM, IC_50_ = 6.72 μM, and SI = 70.4), both plant extracts showed comparable safety and antiviral activities with slightly lower selectivity indices. These data demonstrate that both extracts can be considered as promising candidates for further *in vitro* and *in vivo* studies against SARS-CoV-2 virus and potentially other viruses.

**Table 2:** Selectivity indices (SI) of the tested extracts.

Extract(s)	CC_50_ (μg/ml)	IC_50_ (μg/ml)	SI
*A*. *indica* L. leaves	446.2	8.451	52.8
*M*. *azedarach* L. leaves	351.4	6.922	50.8

Abbreviations: “CC_50_” half maximal cytotoxic concentration; “IC_50_” half maximal inhibitory concentration; “SI” Selectivity index.

To our knowledge, there is a few previous studies that assessed the antiviral activity of *A*. *indica* extracts against the novel SARS-CoV-2 virus [40, 41]. In another virtual study, Baildya *et al*. [42] concluded that, all neem active compounds which extracted from leaves, flowers, seeds and narks have high degree of antiviral activity against Papain-Like Protease (PLpro) of SARS-CoV-2 virus.

Borkotoky and Banerjee [43], isolated five compounds from neem (nimbolin A, nimocin, 7-deacetyl-7-benzoylgedunin, 24-methylenecycloartanol and Cycloeucalenone). These compounds showed high stability and well interacted with crucial regions of E and M proteins of SARS-CoV-2. Moreover, Nimbolin A was the strongest compound can bind free energy with both E and M proteins [43]. Additionally, nimocin and cycloartanols (24-Mmethylenecycloartanol and 24-methylenecycloartan-3-one) also showed strong binding to both E and M proteins [44]. In the same line, Paraiso et al. [45] reported that polyphenols play an important role in alleviating the main SARS-CoV-2 infections in different stages, besides boosting the different organs recovery in post-COVID-19 patients.

In this study, we explored the *in vitro* viral inhibitory activity of *M*. *azedarach* L. leaves extract against SARS-CoV-2 which is so far not studied. Nethmini *et al*. found that *M*. *azedarach* L. could turn off the replication of herpes simplex virus, coxsackie virus, enterovirus, influenza A virus, and bovine rhinovirus in both epithelial and macrophage cell lines [46].

### 3.5. Antibacterial activity

In addition to the *in vitro* anti-SARS-CoV-2 effect in this study for both plant leaves extracts, the inhibitory zone assay was done to assess the *in vitro* antibacterial properties of the two leaves extracts against *E*. *coli*, *P*. *aeruginosa*, *S*. *aureus*, *L*. *monocytogenes*, and *E*. *faecalis* using agar well-diffusion assay. The obtained results indicated that 50 μg/ml of *A*. *indica* L. and *M*. *azedarach* L. displayed high potency for inhibiting a broad spectrum of examined Gram-negative (GN) species (**Table 3)**.

**Table 3:** Antibacterial activity and zone of inhibition (diameter in mm) of the tested plant leaves extracts of *A*. *indica* L. and *M*. *azedarach* L. against selected Gram-negative and Gram-positive bacteria.

Tested extracts	Diffusion Assay	Targeted pathogenic bacteria					
		*E*. *coli*	*S*. *enterica*	*P*. *aeruginosa*	*S*. *aureus*	*L*. *mono*.	*E*. *faecalis*
*A*. *indica* L.	Disc	18 ±0.23	16 ±0.11	15 ±0.11	13 ±0.31	11 ±0.17	12 ±0.31
	Well	21 ±0.18	17 ±0.27	18 ±0.27	16 ±0.28	12 ±0.21	13 ±0.28
*M*. *azedarach* L.	Disc	16 ±0.28	13 ±0.15	12 ±0.15	10 ±0.24	9 ±0.42	10 ±0.24
	Well	18 ±0.21	16 ±0.31	14 ±0.31	12 ±0.29	11 ±0.16	13 ±0.29
**Vanco.**	Disc	9 ±0.11	7 ±0.21	6 ±0.21	12 ±0.22	9 ±0.43	11 ±0.31
	Well	10 ±0.23	9 ±0.24	8 ±0.24	14 ±0.27	11 ±0.11	13 ±0.23
**Cipro.**	Disc	14 ±0.28	11 ±0.15	9 ±0.15	8 ±0.24	6 ±0.25	9 ±0.19
	Well	16 ±0.19	12 ±0.31	11 ±0.31	10 ±0.29	8 ±0.18	10 ±0.15

Simultaneously, two reference antibiotics drugs vancomycin (30 μg/disc) and ciprofloxacin (5 μg/disc) were applied in this study as a positive control (**Table 3**). Results revealed that the size of ZOI of vancomycin against the selected pathogens; *E*. *coli*, *S*. *enterica*, *P*. *aeruginosa*, *S*. *aureus*, *L*. *monocytogenes*, and *E*. *faecalis* using disc diffusion assay were ranged between 7 and 11 mm as mentioned in Table 3. In comparison, the size of ZOI of ciprofloxacin was ranged between 6 and 14 mm (**Table 3**).

These results show the tendency that the GN bacterial species were more susceptible to all tested plant extracts than the GP species. Conversely, the width of ZOI around discs was lower than those around wells. Our data are in agreement with previous study [21], in which the authors demonstrated that the size of ZOI in well-diffusion assay is larger than disc diffusion assay [21]. The ZOI width of vancomycin as a reference drug was less than the ZOI by the tested plant extracts. This indicates that the extracts’ inhibitory efficiency is greater than that of two commonly prescribed antibiotics.

**Fig 3:**
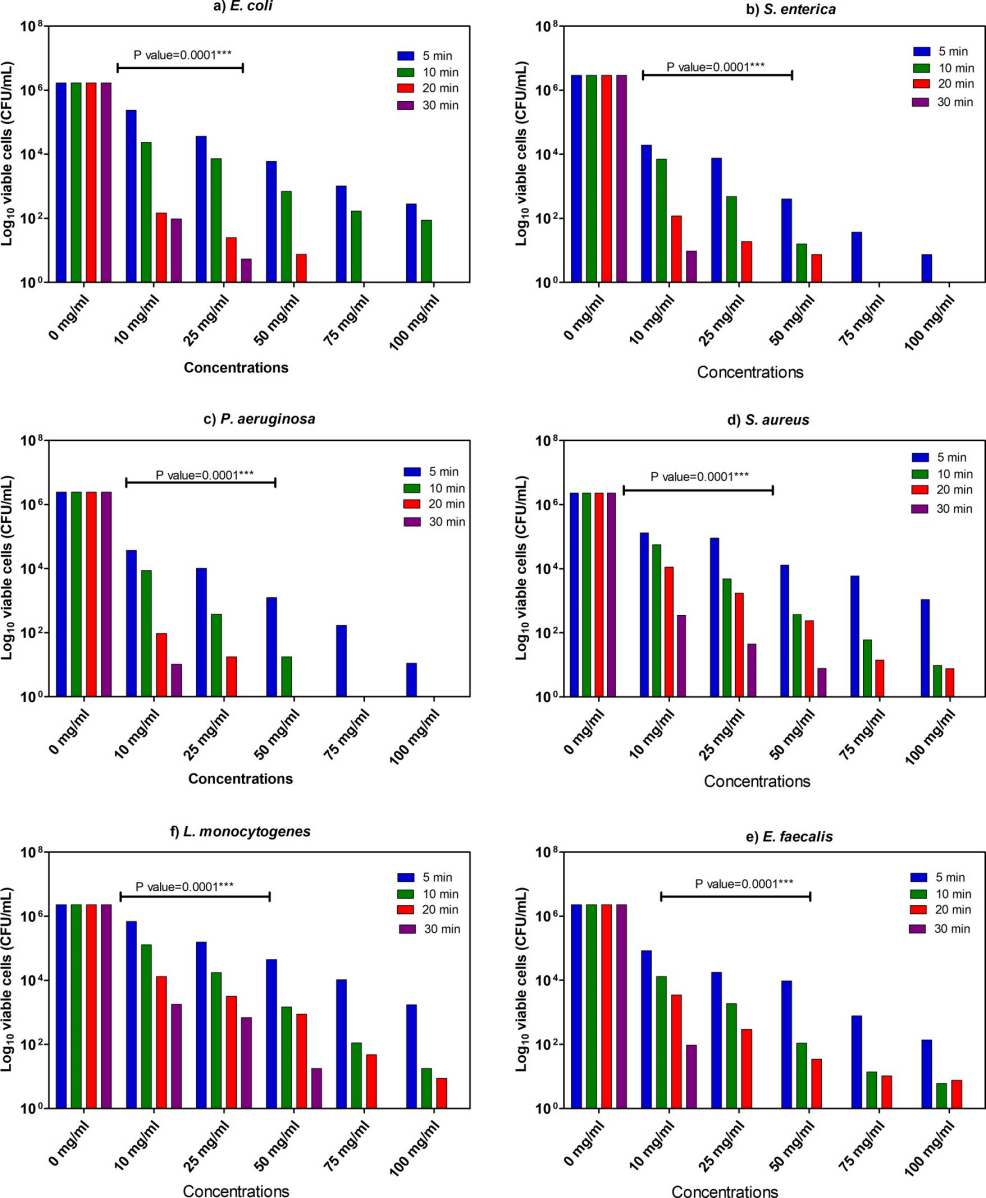
Minimum inhibitory concentration values of methanol extract of *A*. *indica* L. towards examined bacterial species. The remaining viable cell populations after various exposure time intervals of 5, 10, 15, and 30 min to the extracts are also shown. Two-way analysis of variance (*ANOVA*) states ** indicates moderate correlation (*p* ≤ 0.01), *** indicates high correlation (*p* ≤ 0.001).

**Fig 4:**
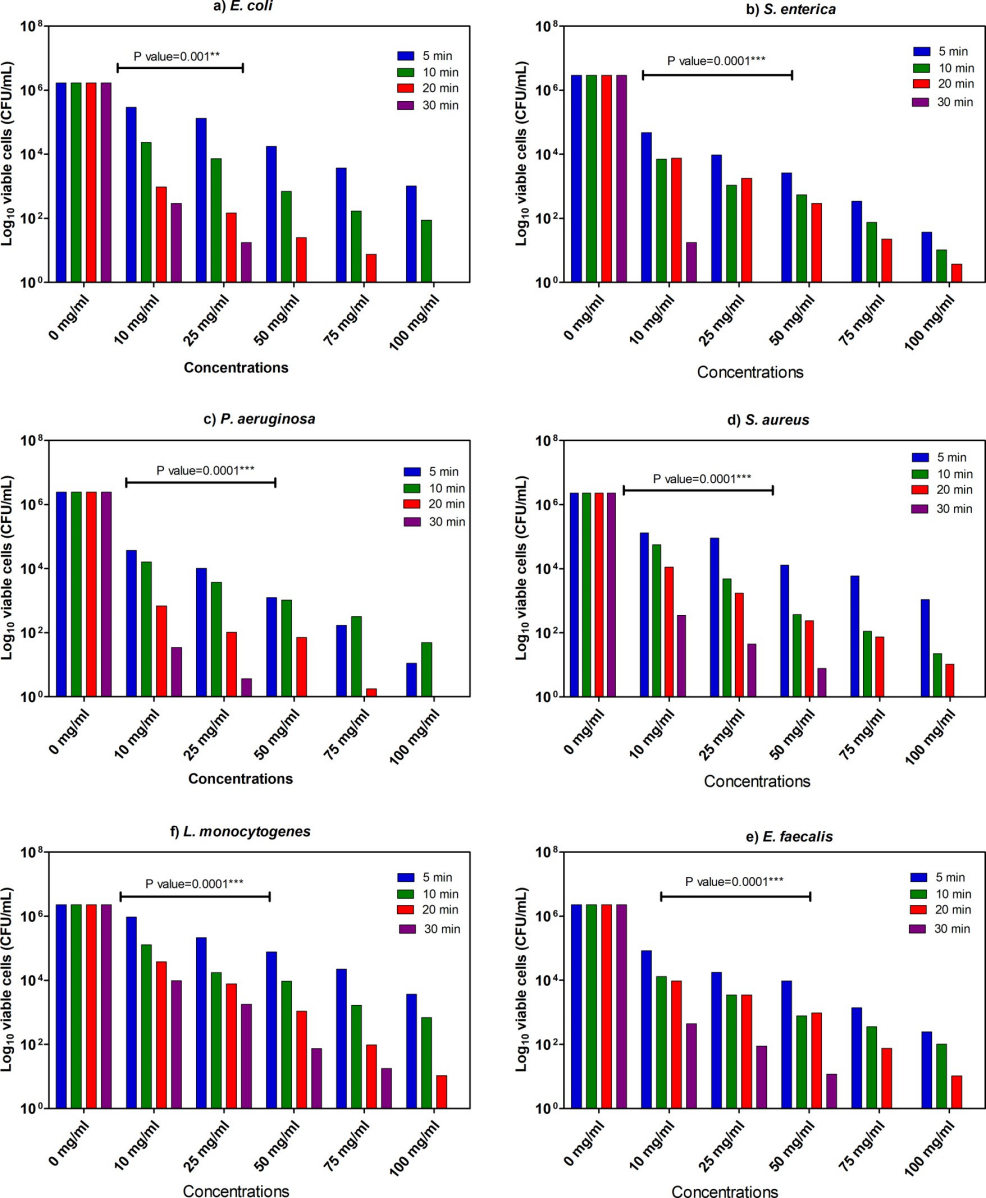
MIC (Minimum inhibitory concentration) values of plant leaves methanol extract of *M*. *azedarach* L. towards examined bacterial species. The remaining viable cell populations at various time intervals of 5, 10, 15, and 30 minutes are also shown. Two-way analysis of variance (*ANOVA*) states ** indicates moderate correlation (*p* ≤ 0.01), *** indicates high correlation (*p* ≤ 0.001).

The different concentrations (10–100 mg/mL) of *A*. *indica* L. and of *M*. *azedarach* L. extracts were examined to estimate MIC values to assess their antibacterial potential during various exposure times (5, 10, 20 and 30 min) to different bacterial strains. As shown in **Figs 3 and 4**, the extracts demonstrated a notable bactericidal effect against all the tested bacterial strains. The detected MIC values depend on the concentrations (mg/mL) and exposure time (min). The consequences of MICs exhibited by the extract of *M*. *azedarach* displayed that the perfect antibacterial effects against *E*. *coli* (MIC = 50 mg/mL within 30 min), *S*. *enterica* (MIC = 25 mg/mL within 30 min), *P*. *aeruginosa* (MIC = 25 mg/mL within 30 min), *S*. *aureus* (MIC = 75 mg/mL within 30 min), *E*. *faecalis* (MIC = 25 mg/mL within 30 min) and *L*. *monocytogenes* (MIC = 75 mg/mL within 30 min). The measured MIC values of *A*. *indica* plant extract were lower in GN than GP bacteria. The enhanced antibacterial activity against GN could be due to variation in active compounds in *M*. *azedarach* extract and their penetration ability through bacterial cell wall and membrane [22]. This finding is encouraging as the variety of treatment options against multi-resistant GN bacteria is highly restricted as compared to GP bacteria where several reserve antibiotics are available.

Using the same procedure and bacterial strains, we also assessed the antibacterial activity of *M*. *azedarach* extract. The results represented in **Fig 4** revealed significant antibacterial effects against all test bacteria. Also here, the calculated MIC values depended on the applied extract concentrations (mg/mL) and contact time (min). The results of MIC exhibited that the extract has clear bactericidal effect against *E*. *coli* (MIC = 50 mg/mL within 30 min), *S*. *enterica* (MIC = 25 mg/mL within 30 min), *P*. *aeruginosa* (MIC = 50 mg/mL within 30 min), *S*. *aureus* (MIC = 75 mg/mL within 30 min), *E*. *faecalis* (MIC = 75 mg/mL within 30 min) and *L*. *monocytogenes* (MIC = 100 mg/mL within 30 min). Interestingly, the results showed that the *M*. *azedarach* extract possess higher antibacterial activities against the tested GN bacteria in comparison to GP bacteria, which is matching with what we detected in case of *A*. *indica* plant extract except that MIC of *Listeria monocytogenes* was higher than *S*. *aureus* and *E*. *faecalis* (**Figs 3 and 4**).

From statistical analysis results, the potential correlation with significance between various concentrations of particular extract towards all the targeted pathogenic bacterial strains was briefly reported in **Table 4**. The results gained revealed that there was an exceptional positive correlation with strong significance between GP and GN species and the concentrations of all plant extracts. On the other hand, results perceived that a moderate correlation had been reported for *E*. *coli*.

**Table 4:** Correlation between different doses of *A*. *indica* and *M*. *azedarach* extracts and log of persisted bacterial cells.

Bacterial species	*A*. *indica* L.			*M*. *azedarach* L.		
	Pearson r	P value	R^2^	Pearson r	P value	R^2^
*E*. *coli*	0.9452	**	0.8971	0.8839	**	0.8142
*S*. *enterica*	0.9806	***	0.9615	0.9786	***	0.9577
*P*. *aeruginosa*	0.9752	**	0.9334	0.9383	**	0.8930
*S*. *aureus*	0.9817	***	0.9773	0.9917	***	0.9800
*L*. *mono*.	0.9892	***	0.9785	0.9917	***	0.9835
*E*. *faecalis*	0.9955	***	0.9911	0.9909	***	0.9820

As a public biohazard with millions of deaths every year, antibiotic resistance emerged recently due to the continuous prescription of improper antibiotics at non-lethal doses. Methicillin-resistant *S*. *aureus*, vancomycin-resistant *enterococci*, and *Mycobacterium tuberculosis* are widely documented as challenging hospital-associated infections to treat with known antibiotics. To this point, medicinal plants are recently applied to provide a supplementary treatment line against these bacterial infections [24]. *M*. *azadirachta* L. and *A*. *indica* L. showed powerful antibacterial activities against various GP and GN species. The Meliaceae family has a substantial percentage of polyphenolic chemicals that could possess oxidative activities. *A*. *indica* L. phytochemicals have such a broad range of biological characteristics, especially antibacterial and cancer-fighting potential [25]. This botanical group has antimicrobial capabilities, particularly towards bacteria that cause ailments with drug tolerance [26]. Further, Jeba Malar and her colleagues described the bactericidal potential of *M*. *azedarach* L. towards numerous types of bacterial pathogens [47]. Limonoids are originated from euphane- or tirucallane-type triterpenoid classes and widely distributed in Meliaceae family. They were reported to have many significant bioactivities such as antibacterial, antiviral, antifungal, and anticancer. Their antimicrobial activity were attributed to the destruction of the cell wall unity and coherence resulting exudation of the substance out of the cell and cell lysis [48].

The ethanolic extract of *M*. *azedarach* L. was established to be biocidal agent against *P*. *aeruginosa*, and *E*. *coli*, the lowest activity was documented in aqueous extract of *M*. *azedarach* against *E*. *coli* (8.5 mm) and *S*. *aureus* (8.2 mm). In previous study, *E*. *coli* showed the lowest sensitivity to methanolic extract (6 mm), and no activity was observed in 10, 20, 30 μg/ml of methanolic extract of *M*. *azedar*ach L. [27]. Previous study proved that *M*. *azedarach* L. seed extracts efficiently manage illnesses caused on by both GP and GN species and reported that the that ethyl acetate fraction exhibited greatest suppression, followed by aqueous and methanol-based extracts, each of which reduced the proliferation of all the studied pathogenic strains. The petrol and benzene extracts displayed antimicrobial activity versus 15 pathogens, however, in comparison to the polarity extracts. The findings previously illustrate that seed extracts are powerful antibiotics that can effectively prohibit both disease-caused pathogens to human infections.

## 4. Conclusions

*A*. *indica* L. and *M*. *azedarach* L. leaves were chemically characterized and were found to be rich in phenolic compounds and flavonoids. *In vitro* assessment of both extracts revealed that they have robust antiviral activity against SARS-CoV-2 as well as antibacterial effects against broader spectra of GN and GP bacteria. These data emphasize their broad-spectrum medicinal value and demand further *in vitro* and *in vivo* investigations as anti-SARS-CoV-2 candidate and antibacterial agents.
